# Tuina for osteoporosis: A systematic review protocol

**DOI:** 10.1097/MD.0000000000009974

**Published:** 2018-02-23

**Authors:** Youkang Dong, Rong Zhao, Chunlin Wang, Taipin Guo

**Affiliations:** a First Affiliated Hospital of Unnan University of Traditional Chinese Medicine/Yunnan Province Hospital of Traditional Chinese Medicine; b School of Acupuncture—Tuina and Rehabilitation, Yunnan University of Traditional Chinese Medicine, Kunming, China.

**Keywords:** osteoporosis, protocol, systematic review, tuina

## Abstract

**Background:**

Osteoporosis is one kind of commonly and frequently occurring global disease accompanying with serious complications. As a branch of the subject of Acupuncture-Tuina, tuina is widely applied for osteoporosis alone or combined with other methods in China and other nations while its effective evidence is not clear. Hence, this systematic review protocol purpose is to evaluate the value of its efficacy and safety for osteoporosis.

**Methods:**

This systematic review and meta-analysis will be performed by means of electronic databases including Cochrane Library, Medline, Cochrane Library, Web of Science, EBASE, Springer, WHO International Clinical Trials Registry Platform (ICTRP), China National Knowledge Infrastructure (CNKI), Wanfang database, Chinese Biomedical Literature Database (CBM), Chinese Scientific Journal Database (VIP) and others with valid search strategy probably. The assessment of bias risk, data synthesis, subgroup analysis, and meta-analyses will be conducted using RevMan V.5.3.5 software.

**Results:**

This systematic review will present a high-quality evidence for clinicians and might be the first to evaluate the efficacy and safety of tuina for osteoporosis including alleviation of pain, adverse event, spinal motor function improvement as well as improvement of self-care ability and daily living.

**Conclusion:**

This protocol will determine whether or not tuina is an effective and safety intervention for osteoporosis.

## 1 Introduction

Osteoporosis (OP) is characterized by progressive declination in the density of bones and microstructure damage of bone structure, which tends to increase the risk of fractures. And it can be commonly and frequently seen in postmenopausal women as well as elder senior citizens, the prevalence of OP is 10.3% in US older adults among 10.2 million people,^[1]^ and 6.6% in female, 22.1% in male, respectively, aged 50 years or more while 5.5% of total population in the European Union including 27 nations with 27.5 million inflicting from OP.^[2]^ The data in China mainland is about 13.0% of the population in general.^[3,4]^

As the aging population growing fast, the increase of these quantities are going to change correspondingly. The main complication of OP manifested as localized back pain, deformation of spinal column, functional spinal activity disturbance, and fragile fracture of femoral neck and vertebrae, which burden the families and societies financially. The fracture from OP ranked sixth surpass hypertension according to the burden disease of disability adjusted life years (DALY).^[5]^ In postmenopausal OP, the losses of DALY are so considerable that the cost of incident and prior fragility fractures tack up 37 billion Euros of 27 countries in the European in 2010, and it will rise by 25% in 2015 accompanying with the proceeding of aging population.^[6,4]^ The prevention and treatment of OP contains those of basic therapies, lifestyle changes and drugs, which aim to restrain the osteopenia, alleviate the pain, enforce the strength and enhance the quality of the bone which is benefit for lowering the risk of fracture.^[7]^ Although these methods have been used widely in clinic for decades, many inevitable and uncontrollable hazards such as the increase risk in cardio-cerebrovascular events, gastrointestinal reaction, nephrotoxicity, and liver damage happened. Avoiding the occurrence of related events, complementary and alternative therapy such as tuina, acupuncture and Chinese medicine had been applied extensively in treating OP in China.^[8–10]^

Tuina is a kind of physical therapy method guided by Traditional Chinese Medicine (TCM) theories, which is called massage before the Ming dynasty and its history can be traced back to thousand years ago. Nowadays, as a branch of the subject of acupuncture and tuina, it is recorded that tuina can increase the levels of bone mineral density (MBD) and estradiol (E2), alleviate the clinical symptoms of OP in postmenopausal women effectively with few side effects.^[11]^ And it can relieve low-back pain and joint pain as well as improve knee function and metabolic markers.^[12]^ Although abundant of clinical studies existed, there is still controversial for its therapeutic effect owing to worrying about the risk of fracture after tuina especially in serve OP, and the lacks of evidence-based medical (EBM) system evaluation of tuina for OP might be the most important reasons. Therefore, to serve tuina as intervention and OP as object, this study intends to evaluate the efficacy and safety of tuina on OP by means of statistical analysis, and it is very essential of the clinical decision scheme for clinicians.

## 2 Methods

This protocol has been registered in the PROSPERO international prospective register of systematic reviews (CRD42018085204), and was performed in accordance with the preferred reporting items for systematic reviews and meta-analysis protocol (PRISMA-P).^[13]^ This is a literature-based study, ethical approval is unnecessary.

### 2.1 Literature search strategy and study selection

**Figure 1:**
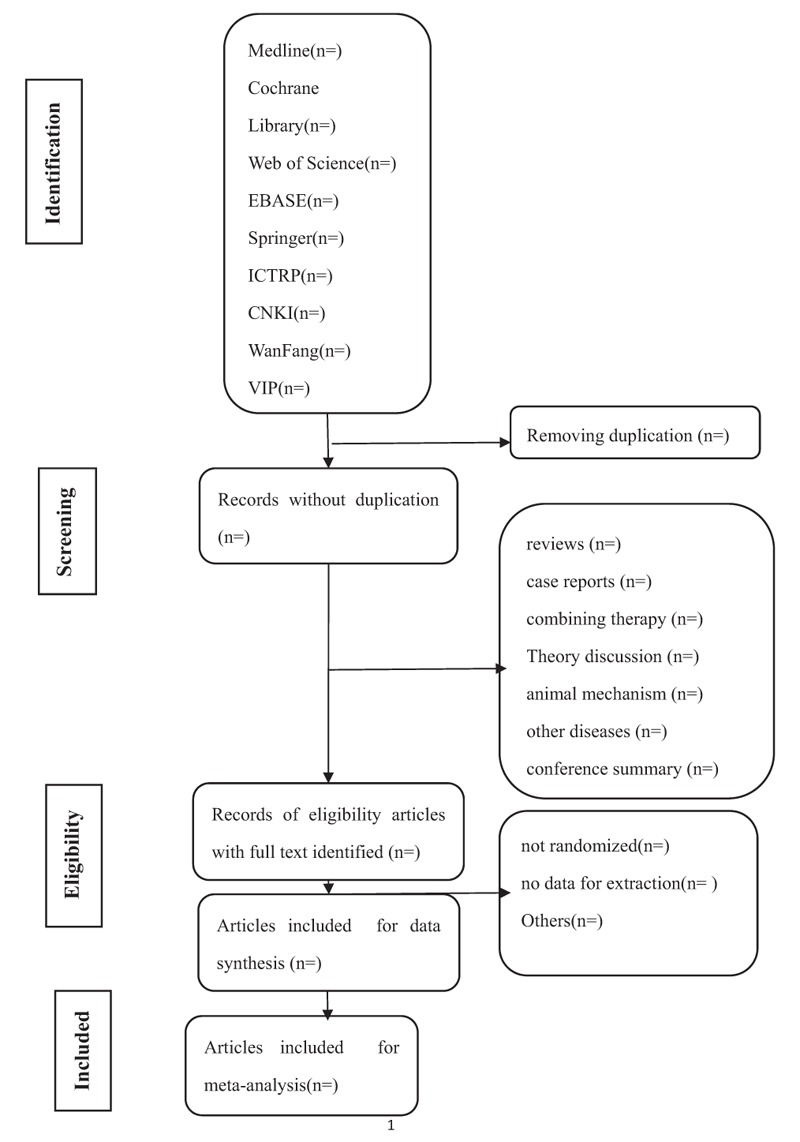
Flow diagram of studies identified.

We will perform a comprehensive literature search of relevant databases including those of Web of Science, Springer, Medline, Cochrane Library, EBASE, Wanfang, Chinese Biomedical Literature Database (CBM), WHO International Clinical Trials Registry Platform (ICTRP), Chinese Scientific Journal Database (VIP), and China National Knowledge Infrastructure (CNKI) from the publishment to January, 2018. The search strategy will be enacted according to the guidance offered from the Cochrane Handbook with the following Medical Subject Heading (MeSH) terms and variants: “osteoporosis,” “osteopenia,” “bone mass loss,” “tuina,” “massage,” “Chinese massage,” “Chinese manipulation,” “maasge therapy,” and all possible spellings of “osteoporosis” and “tuina” (Table 1). The search strategy of Web of Science is listed in Table 1, and others will be modified according to the requirement. All of the studied will be selected and confirmed by Youkang Dong and Rong Zhao, and the relevant literatures will be picked out by perusing titles and abstracts of the primary documents. Literatures that are not conformed to the inclusion criteria will be excluded. If the integrality of article is incomplete, we will resort to the corresponding author or the first author. Any disagreement should be disposed by discussion. The details of the selection process are shown in Fig. 1.

**Table 1:**
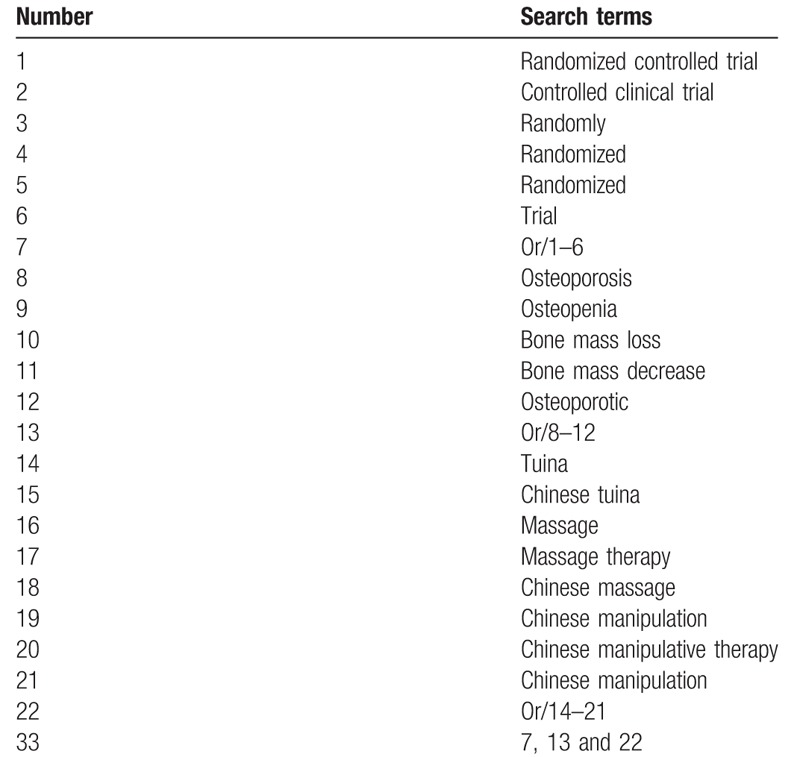
Web of science search strategy.

### 2.2 Outcome measures

This protocol proposes to assess the value of tuina for OP by using the primary outcomes of pain intensity, bone mineral density, and global assessment of improvement proportion; the secondary outcomes of quality of life, biochemical indicators related to OP such as oestradiol (E2), serum calcium (Ca), phosphorus (P), bone Gla protein (BGP), alkaline phosphatase (ALP), calcitonin (CT), parathyroid hormone (PTH), interleukin-6 (IL-6) as well as side effects caused by tuina.

### 2.3 Data extraction

Using an electronic form, the substantial contents of each selected article will be extracted, respectively, by Chunlin Wang and Rong Zhao, the information should be consisted of those items: the name of the first author or correspondence, publication time, design of study which contains blinding, allocation concealment, randomization and case report, the inclusion and exclusion criteria, gender, age, duration of getting OP and other details such as courses of treatment, outcomes, follow-up, side effect even if accident and so on. While the third reviewer named Taipin Guo will recheck data again, we will appeal to the authors proactively to integrate the study information provided that there is information loss. Disagreement will be settled after consulting experts and arbiter.

### 2.4 Quality assessment

Seven items related to the bias risk including generation of random sequences, allocation concealment, blinding, incomplete outcome data, selective reporting, and other issues will be assessed by Taipin Guo and Chunlin Wang, respectively, in accordance with systematic reviews for interventions of Cochrane handbook. Moreover, these items should be judged by low risk of bias, high risk of bias, or unclear risk of bias. While the 2 reviewers mentioned above will appraised the result by means of Grading of Recommendations Assessment, Development and Evaluation (GRADE).^[14,15]^ The disagreement will be settled by discussion.

### 2.5 Data synthesis

Cochrane Collaboration Review Manager software (RevMan V.5.3.5) will be conducted as tool dealing with the data from primary and second studies. Risk ratio (RR) will be analyzed and 95% confidence intervals (CI) presented in dichotomous variable while standard mean difference (SMD) 95% CI for continuous variable. *I*^2^ statistic and χ^2^ test will be applied to assess heterogeneity in the studies with the judgment standard of *I*^2^ will be: below 50% means low heterogeneity; between 50 and 75% means moderate heterogeneity; over 75% means high heterogeneity. A random-effects model and DerSimonian–Laird method will be used calculating the effect estimates When *I*^2^ is over 50% while a fixed-effects one with the Mantel–Haenszel method as *I*^2^ below 50%. As for the trails reported the results before and after the intervention, the mean change will be calculated by the special formula^[16]^ as 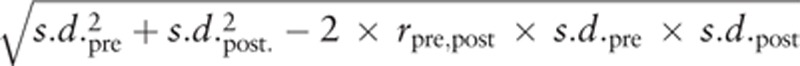, in which take 0.5 as the correlation coefficient (*r*_pre,post_). Additionally, when the outcome is unsuitable to analysis quantitatively, qualitative description will be adopted.

### 2.6 Subgroup analysis

Owing to differentiation of situation such as gender, age, courses of the treatment, disease condition, race, kinds of tuina intervention, we will conduct subgroup analysis base on the data.

### 2.7 Sensitivity analysis

If there are sufficient data available, the sensitivity analysis will be carried out evaluating the effect of single studies on the whole estimate by excluding studies of integration (e.g., tuina, acupuncture, medicine, and others) treating OP.

### 2.8 Ethics and dissemination

This systematic review has no requirement of ethical approval and informed consent, and the result will be disseminated as a literature review and conference for the clinician.

## 3 Discussion

As a kind of commonly and frequently global disease in the senile individuals and postmenopause women, osteoporosis causes a great deal of burden financially and socially. Unfortunately, there is no acknowledged approaches from the Evidence-based medical (EBM) of treatment and prevention dealing with this problem.

Being a technique, tuina plays a significant role in treating and preventing disease as with acupuncture in the TCM hospitals or clinics, and as a complementary for OP, it has been used years with the efficacy of promoting blood circulation, nourishing the function of nerve and immune system, alleviate spasm and pain, dredging the meridian and channel as well, which is conformed to the mechanism with kidney deficiency and blood stasis of OP from TCM theory.^[17–19]^ However, up to now, there has no relevant systematic review been reported.

This might be the first time to analyze literature with systematic review on the whole of tuina for OP, the purpose of this systemic review and meta-analysis is to synthesize the existing trials of tuina at home and abroad for OP using the method of EBM, aims to offer a forceful proof of effectiveness and safety for clinical practice, scientific researchers and health policymakers.
