# Tuina for osteoporosis: A systematic review protocol: Erratum

**DOI:** 10.1097/MD.0000000000011672

**Published:** 2018-07-20

In the article, “Tuina for osteoporosis: A systematic review protocol”,^[1]^ which appeared in Volume 97, Issue 8 of *Medicine,* affiliation a should appear as “The First Affiliated Hospital of Yunnan University of Traditional Chinese Medicine/Yunnan Province Hospital of Traditional Chinese Medicine.”
